# Lymphomatoid papulosis misdiagnosed as pityriasis lichenoides et varioliformis acuta: Two case reports and a literature review

**DOI:** 10.3892/etm.2014.2006

**Published:** 2014-10-07

**Authors:** YAN ZHENG, JINJING JIA, QIONG TIAN, XINYU DONG, XIN WANG, ZHAOXIA YING, SHENGXIANG XIAO, WENSHENG LI

**Affiliations:** 1 Department of Dermatology, The Second Affiliated Hospital of Xi’an Jiaotong University, Xi’an, Shaanxi 710004, P.R. China; 2 Department of Pathology, The Third Affiliated Hospital of Xi’an Jiaotong University, Xi’an, Shaanxi 710068, P.R. China

**Keywords:** primary skin, CD30^+^ lymphoproliferative disorders, lymphomatoid papulosis, pityriasis lichenoides et varioliformis acuta

## Abstract

The aim of this study was to improve the level of diagnosis and differential diagnosis of lymphomatoid papulosis (LyP). Two cases of type B LyP were identified and the literature was reviewed to summarize the clinical outcomes and pathology of LyP and its treatment. The two patients exhibited symptoms with papulonodular lesions, the centers of which gradually underwent ulceration and necrosis. CD30, a helper T-cell marker specifically expressed in tumor cells was analyzed by immunohistochemical staining and the result showed that CD30-negative or only scattered CD30-positive cells were present. Therefore, a diagnosis of type B LyP was made. A fairly good curative effect was achieved following treatment with retinoic acid, glucocorticoids and immunomodulatory drugs. LyP is a type of low-level malignant lymphoma and is easily misdiagnosed as pityriasis lichenoides et varioliformis acuta and other diseases. In order to avoid under diagnosis and misdiagnosis, doctors should evaluate suspected patients by histopathological and immunohistochemical examination.

## Introduction

Lymphomatoid papulosis (LyP) is a chronic papulonecrotic or papulonodular skin disease with histological features suggestive of a malignant lymphoma (1). The disease is characterized by recurrent crops of pruritic papules, predominantly on the trunk and limbs, at different stages of development. The papules heal spontaneously over 1–2 months, usually leaving slightly depressed oval scars (2–4). The clinical manifestation of the disease is similar to that of pityriasis lichenoides et varioliformis acuta; as a result, LyP is easily misdiagnosed. The diagnosis is primarily dependent on the histopathology and immunohistochemical examination. The present study reports two cases of LyP confirmed by the Second Affiliated Hospital of Xi’an Jiaotong University (Xi’an, China), with a literature review. The study was approved by the Ethics Committee of the Second Affiliated Hospital of Xi’an Jiaotong University. Written informed consent was provided by the patients involved.

## Case reports

### Case 1

A 17-year old male who had multiple papules and nodules with necrosis on the trunk and limbs that had persisted for one month was admitted to the Second Affiliated Hospital of Xi’an Jiaotong University. One month prior to admission, no evident red papules or nodules were visible on the torso and limbs. However, papules and nodules measuring 0.8×0.4 cm had started to appear, which gradually increased in number, developed an ulcerated, crusted surface and were slightly itchy but painless. Accordingly, this case was initially diagnosed as mite dermatitis at another hospital. As prescribed, the patient applied compound indomethacin tincture and mometasone furoate cream to the skin twice a day for two weeks. However, the curative effect was poor. Subsequently, in the Second Affiliated Hospital of Xi’an Jiaotong University, this case was diagnosed as pityriasis lichenoides et varioliformis acuta and the patient was treated with an antibiotic (clarithromycin, 0.5 g orally once a day for one week), and corticosteroid cream (twice a day for two weeks). Histopathological and immunohistochemical examination was also performed.

From the onset of the disease, the patient remained normal in spirit, food intake, night rest and urinary function, and underwent no significant changes in body weight. When he was two years old, the patient suffered from urticaria with severe itch, and following treatment with calamine lotion, the rash subsided. Every two years subsequent to this, similar urticarial lesions recurred in the summer or autumn. The symptoms were relieved following the administration of astemizole and calamine lotion. The patient’s grandfather was affected by psoriasis. Other than this, the patient was unaware of other genetic disorders in his family history.

**Figure 1:**
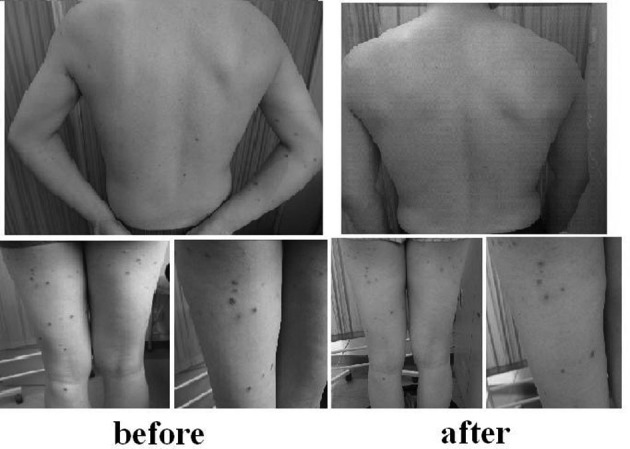
Clinical features of case 1 before and after treatment.

Physical examination revealed that the patient had stable vital signs, and his superficial lymph nodes were not palpably enlarged. In addition, a physical examination revealed no abnormalities, with the exception of the large red papules and nodules, 0.2–1.0 cm in diameter, that were scattered on the trunk and limbs. Parts of these exhibited necrosis, ulceration, overlying black crusts in the center and white scales on the surface. Old lesions were visible as atrophic scars of various sizes with different degrees of pigmentation. When palpated, the lesions were hard and not tender (Fig. 1).

**Figure 2:**
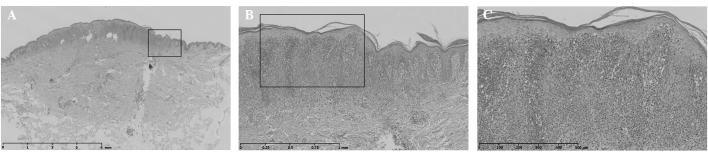
Histopathological examination of case 1 by hematoxylin and eosin staining. (A) Hyperkeratosis of the epidermis, parakeratosis, acanthosis and dermal edema are exhibited. Dense, band-like lymphocytic infiltration was observed and atypical lymphocytes were infiltrating into the epidermis (scale bar length = 4 mm). (B) Enlarged view of A (scale bar length = 1 mm). (C) Enlarged view of B (scale bar length = 500 μm). The papillary layer of the corium showed clear edema. The red blood cells appeared to show extravasation. Scattered lymph cells infiltrated in the mid-dermis. Certain lymphocytes showed nuclear invagination with cerebriform nuclei.

Histopathological examination showed that the patient had hyperkeratosis of the epidermis, dyskeratosis, acanthosis and dermal edema. In addition, dense, band-like lymphocytic infiltration was observed and a large number of atypical lymphocytes had infiltrated into the epidermis. The papillary layer of the dermis showed evident edema. Red blood cells appeared to be undergoing extravasation. Scattered malignant lymph cells infiltrated into the mid-dermis. Certain lymphocytes showed nuclear invagination with cerebriform nuclei (Fig. 2).

**Figure 3:**
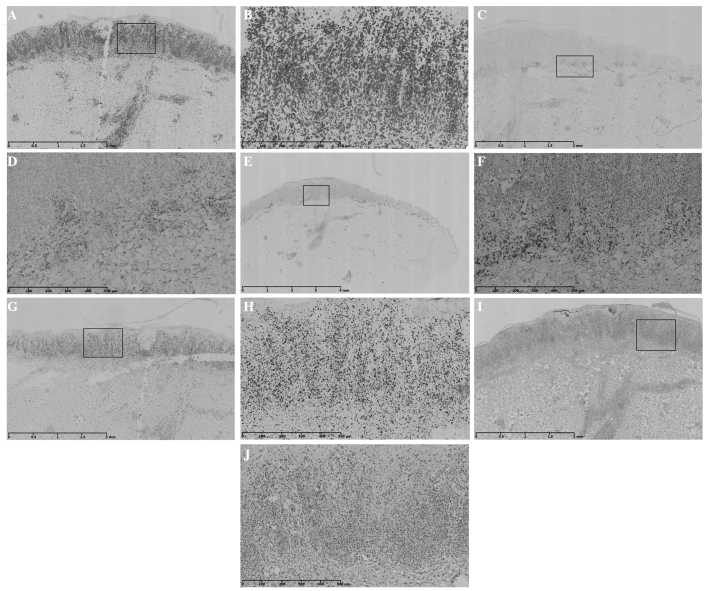
Immunohistochemical staining of case 1 shows positive or partly positive results for (A and B) CD3, (C and D) CD4, (E and F) CD45RO, (G and H) Ki67 and (I and J) TIA-1. (B, D, F, H and J) are magnified images (scale bar length = 500 μm) of (A, C, E, G and I), respectively. Scale bar length: (A, C, G and I) 2 mm and (E) 4 mm. CD, cluster of differentiation; TIA-1, T-cell intracellular antigen-1.

**Figure 4:**
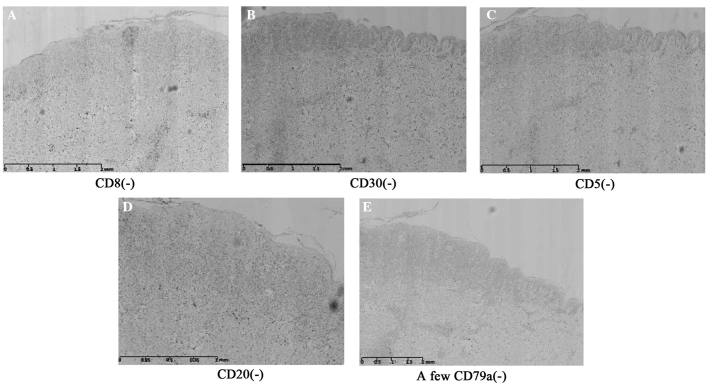
Immunohistochemical staining of case 1 samples show negative results for (A) CD8, (B) CD30, (C) CD5, (D) CD20 and (E) CD79a. Scale bar length: (A, B and D) 2 mm and (C and E) 1 mm. CD, cluster of differentiation.

Immunohistochemical staining demonstrated that this case was cluster of differentiation (CD)3-, CD4- and T-cell intracellular antigen-1 (TIA-1)-positive. The Ki67-positive rate was 70% and CD45RO was partially positive (Fig. 3). In addition, the case was CD5-, CD8-, CD20- and CD30-negative and a small amount of CD79a was observed, which was considered negative (Fig. 4). Accordingly, the patient was diagnosed with LyP of the type B/mycosis fungoides (MF) type in histopathology. The patient was treated with Viaminate capsules (25 mg, three times a day for one month), Transfer Factor capsules (25 mg, three times a day for one month) and disodium glycyrrhizinate (0.1 g, twice a day for one month) orally, and glucocorticoid cream for external use (once a day for two weeks). Subsequent to the treatment, the lesions gradually subsided and no new lesions formed (Fig. 1).

### Case 2

A 30-year-old male patient was admitted to the Second Affiliated Hospital of Xi’an Jiaotong University exhibiting nodules in the left waist that had been present for three months, spreading over the whole body with ulceration and scab formation for two months. Three months prior to admission, three purple-red papules measuring 0.8×0.4 cm appeared in the left waist without significant external stimuli, which did not bother the patient as there were no clear symptoms. A month subsequent to this, similar papules and nodules gradually appeared over the patient’s whole body. They increased in number, ulcerated, generated an exudate, and had a putrescent and scabbed central section. Some of the old lesions spontaneously subsided, leaving blackish-brown pigmentation. Concurrently, new lesions appeared that were painless but mildly itchy. The local clinic diagnosed this case as herpes and treated the patient with acyclovir for one month. However, the curative effect was poor. Thus, in the hospital, this case was diagnosed as pityriasis lichenoides et varioliformis acuta and the patient was orally treated with clarithromcin (0.5 g, once a day), levocetirizine hydrochloride tablets (5 mg, once a day) and compound glycyrrhizin capsules (50 mg, three times a day) for one week. Histopathological and immunohistochemical examination was also conducted.

**Figure 5:**
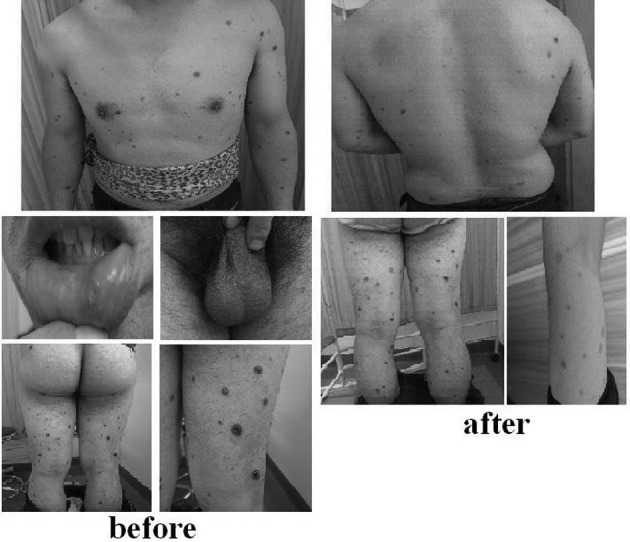
Clinical features of case 2 before and after treatment.

From the onset of the disease, the patient remained normal in spirit, food intake, night rest and urinary function and no significant changes in body weight occurred. The patient had no history of severe disease and was not allergic to any food or drugs. His father had succumbed to esophageal cancer and the patient was unaware of any similar disease or other genetic disorders in his family history. The results from a physical examination revealed that the patient had stable vital signs and superficial lymph nodes that were not palpably enlarged. No abnormality of the heart, lungs or abdomen was identified. A number of red papules and nodules appeared on the face, neck, trunk, limbs, lip mucosa and scrotum of the patient. These were 0.2–2.0 cm in diameter and contained sections that exhibited necrosis and overlying central black crusts. Old lesions formed atrophic scars of various sizes and with different degrees of pigmentation, sections of which were slightly atrophied. When palpated, the lesions felt hard and were not tender (Fig. 5).

**Figure 6:**
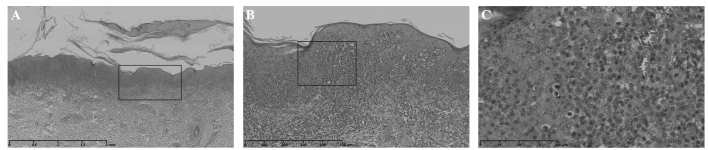
Histopathological examination of case 2 by hematoxylin and eosin staining. (A) Hyperkeratosis of the epidermis, dyskeratosis, acanthosis and dermal edema are exhibited. Dense, band-like lymphocytic infiltration was observed and atypical lymphocytes were infiltrating into the epidermis (scale bar length = 2 mm). (B) Enlarged view of A (scale bar length = 500 μm). (C) Enlarged view of B (scale bar length = 100 μm). The papillary layer of the corium showed clear edema. The red blood cells appeared to show extravasation. Scattered lymph cells infiltrated in the mid-dermis. Some of the lymphocytes had nuclear invagination with cerebriform nuclei.

Histopathological examination revealed hyperkeratosis of the epidermis, dyskeratosis, acanthosis and dermal edema. Dense lymphocytic infiltration was observed in bands, and numerous atypical lymphocytes had infiltrated into the epidermis. The papillary layer of the dermis exhibited evident edema. Extravasation of the red blood cells was also apparent. Scattered malignant lymph cells infiltrated into the mid-dermis and some lymphocytes had invaginated, cerebriform nuclei (Fig. 6).

**Figure 7:**
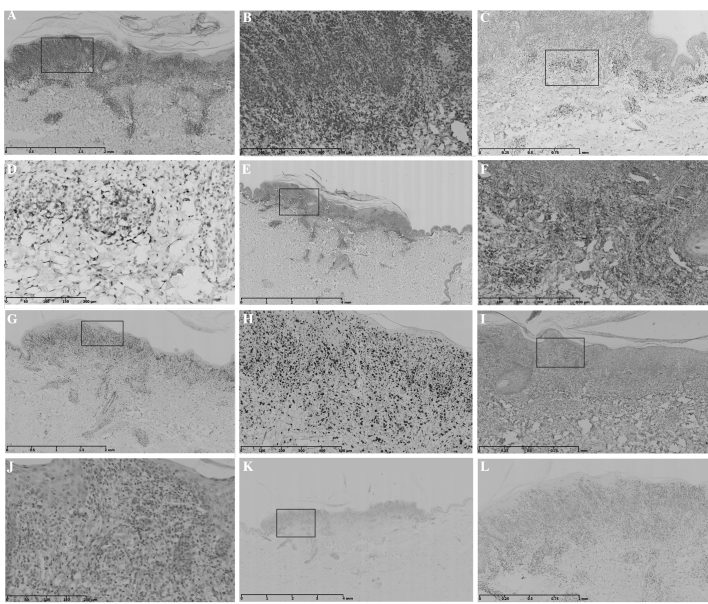
Immunohistochemical staining of case 2 shows positive or partly positive results for (A) CD3, (C) CD5, (E) CD45RO, (G) Ki67, (I) CD30 and (K) anaplastic lymphoma kinase. (B, D, F, H, J and L) are magnified images of (A, C, E, G, I and K), respectively. Scale bar length: (A) 2 mm; (B) 500 μm; (C) 1 mm; (D) 200 μm; (E) 4 mm; (F) 500 μm; (G) 2 mm; (H) 500 μm; (I) 1 mm; (J) 200 μm; (K) 4 mm; (L) 1 mm. CD, cluster of differentiation.

**Figure 8:**
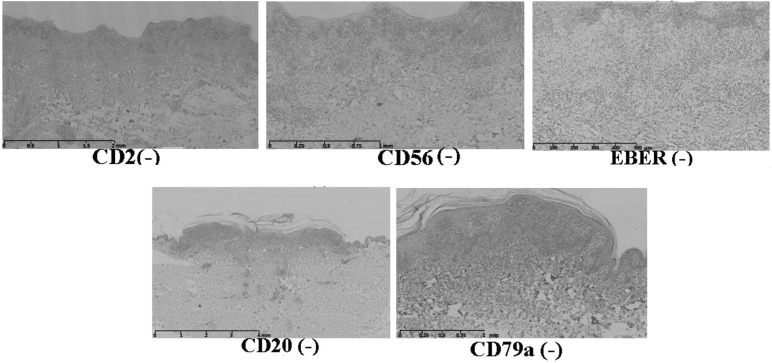
Immunohistochemical stainings of case 2 shows negative results for (A) CD2, (B) CD56, (C) Epstein-Barr virus-encoded RNA, (D) CD20 and (E) CD79a. Scale bar length: (A) 2 mm; (B) 2 mm; (C) 500 μm; (D) 4 mm; (E) 1 mm. CD, cluster of differentiation.

Immunohistochemical staining demonstrated that this case was CD3-, CD5-, CD45RO- and anaplastic lymphoma kinase (ALK)-positive, a few scattered large cells were CD30-positive and 40–50% of cells were Ki67-positive (Fig. 7). In addition, the case was CD2-, CD20-, CD79a-, CD56- and Epstein-Barr (EB) virus-encoded RNA (EBER)-negative (Fig. 8). Accordingly, the patient was diagnosed with LyP type B. The patient was treated with methylprednisolone tablets (4 mg, once every morning) and Viaminate capsules (25 mg, three times a day) orally, desonide cream (twice a day) and compound polymyxin B ointment for external use (once a day) topically, and intramuscular mannatide injection (5 mg, once every alternate day) for one month. Following the treatment, the papules shrank and the ulcerated nodules became crusted, flattened and gradually subsided. The original pigmentation faded and no new lesions formed (Fig. 5).

## Discussion

The term LyP was originally used by Macaulay (5) in 1968 to describe ‘a self-healing rhythmical paradoxical eruption, histologically malignant but clinically benign’. However, the classification system for cutaneous lymphomas has evolved rapidly. During consensus meetings in 2003–2004, the World Health Organization-European Organization for Research and Treatment of Cancer (WHO-EORTC) classification grouped LyP among the indolent cutaneous T-cell lymphomas (6,7). LyP is classified as a cutaneous lymphoma due to its association with other malignant lymphoproliferative disorders. LyP is part of a spectrum of CD30-positive cutaneous lymphoproliferative diseases that also includes primary cutaneous anaplastic large cell lymphoma (ALCL) and borderline CD30^+^ lesions (8,9). The etiology and pathogenesis of LyP remain unclear, but have been speculated to have an association with infections by paramyxovirus, human leukemia/lymphoma virus or EB virus (10–13).

LyP can be found in individuals of any age, but most commonly occurs in adults, particularly those aged >40 years, with no significant difference between genders (14,15). It is mainly divided into three types in the clinical setting. These are the classic type (type A), an MF-like type in which plaque parapsoriasis or MF-like plaques are present (type B), and a limited granuloma type, which is similar to Hodgkin’s disease (type C). Since the 1980s, several subtypes have been reported, including LyP of hair follicles, eosinophilic histiocytosis and hypereosinophilic syndrome (16,17). The lesions frequently occur on the trunk and proximal extremities and may also be found on the palms and soles, scalp and the genital and oral mucosa. LyP is similar to pityriasis lichenoides et varioliformis auta clinically and its characteristics include skin lesions that appear in groups, reddish, purple or red-brown papules and recurring nodules that appear in small or large numbers (up to several hundreds) and distribute symmetrically with unequal size (typically <2 cm). A few papules develop into vesicles and pustules, and they can also develop into masses with diameters of 5–15 cm, followed by gradual necrosis, ulceration, or the formation of a scab or surface scaliness (18). In general, single lesions regress in 3–12 weeks, leaving hyperpigmentation or atrophic scars. Since LyP is chronic and skin lesions persist in recurring and fading, lesions of various types may be present during the same period of time. Lesions confined to a region are known as limited type LyP, which is rare in clinical practice (19). Lymphadenopathy, thyroiditis, fever, weight loss and fatigue may also be associated with LyP (20).

The 2005 WHO-EORTC cutaneous lymphoma classification divided LyP into types A, B and C, but there are no strict boundaries separating these types from each other. Occasionally, individual lesions may combine A, B or C types, which is termed a hybrid. In type A, or the histiocytic type, specimens display dense mixed infiltrates characterized by large atypical lymphocytes and neutrophils, eosinophils, histiocytes and small lymphocytes. The large atypical cells do not form sheets or constitute >50% of the infiltrating cells. In type B, or the lymphocytic type, specimens display a monomorphous infiltrate of small to medium-sized lymphocytes with cerebriform nuclei similar to those observed in MF. This variant has also been referred to as MF-like LyP in the literature (21). In type C, specimens display cytological features similar to those of type A and ALCL, and the atypical lymphoid cells either form sheets or large nodules and these represent >50% of the infiltrating cells. This type has been referred to as borderline LyP-ALCL (22).

Atypical cells of the three types described above can express proteins associated with cytotoxic T cells, including TIA-1, perforin and granzyme B (GrB). These cells are mostly ALK-negative, but few of them are positive. They are usually epithelial membrane antigen- and CD15-negative. The percentage of the total cells that are Ki67-positive is 50–95% (23).

In addition to these three previously known types, a novel type of LyP, designated type D, was recently proposed (24,25). Although the clinical presentation of type D LyP is similar to that of CD8-positive cytotoxic T-cell lymphoma, type D LyP has a more favorable prognosis. These conditions differ in that CD30 expression in LyP type D is positive. Thus, the clinical diagnosis must be conducted carefully to identify the condition correctly.

Since LyP is self-limiting and the prognosis is promising, with the exception of a few lesions that do not heal well, and lesions in the face, hand, foot and genitals causing aesthetic, function and scarring problems, the majority of patients do not require active treatment. The current reported treatment methods are the systemic or local application of glucocorticoids, phototherapy (psoralen and ultraviolet A light, ultraviolet B treatment and photodynamic therapy), retinoic acid, methotrexate, interferon, radiotherapy, nitrogen mustard and hormone replacement therapies (26–31). The five-year survival rate is 100% (32,33). However, a number of studies have found that 10–20% of patients may exhibit diseases prior to, coexisting with or subsequent to LyP, including MF, Hodgkin’s lymphoma, ALCL or other tumors. These diseases have a high chance of developing into malignant lymphoma; therefore, it is recommended that all patients with LyP should be followed-up throughout their lifetime (34–38).

The two cases described in this paper involve typical skin lesions, characterized by multiple papules and nodules with gradually broken and scabbed centers. Since the clinical manifestation is similar to that of pityriasis lichenoides et varioliformis acuta, it is easily misdiagnosed. Therefore, histopathological and immunohistochemical examinations were conducted and finally the two patients were diagnosed with type B LyP. The treatments prescribed to the two patients included retinoic acid and immunomodulatory drugs taken orally and corticosteroid ointment for external use. In view of the increased severity of case 2, a small dose of hormone orally and antibiotic for external use was included. The two patients obtained a satisfactory curative effect. As Lyp may be secondary to other malignant tumors, and due to the short duration of follow-up, close observation of the patients remains necessary in the future.
